# Defining well-being in psoriasis: A Delphi consensus among healthcare professionals and patients

**DOI:** 10.1038/s41598-024-64738-6

**Published:** 2024-06-24

**Authors:** Esteban Daudén, I. Belinchón, E. Colominas-González, P. Coto, P. de la Cueva, F. Gallardo, J. L. Poveda, E. Ramírez, S. Ros, R. Ruíz-Villaverde, M. Comellas, Luís Lizán

**Affiliations:** 1 Department of Dermatology, Instituto de Investigación Sanitaria de La Princesa (IIS-IP), Hospital Universitario de La Princesa, Diego de León, 62, 28006 Madrid, Spain; 2 Department of Dermatology, Hospital General Universitario Dr Balmis-ISABIAL-UMH, Alicante, Spain; 3 Department of Pharmacy, Hospital del Mar, Barcelona, Spain; 4 Department of Dermatology, Hospital Vital Álvarez Buylla, Mieres, Spain; 5 Department of Dermatology, Hospital Universitario Infanta Leonor, Madrid, Spain; 6 Department of Dermatology, Hospital del Mar, Barcelona, Spain; 7 Department of Pharmacy, Hospital Universitario y Politécnico La Fe, Valencia, Spain; 8 Department of Pharmacy, Hospital Universitario de La Princesa, Madrid, Spain; 9 Departments of Dermatology and Rheumatology, and Cardiac Transplant Unit, Hospital de la Santa Creu i Sant Pau, Barcelona, Spain; 10 Department of Dermatology, Hospital Universitario San Cecilio, Granada, Spain; 11 Outcomes’10, Castellón de la Plana, Spain; 12 Outcomes’10, Universidad Jaume I, Castellón de la Plana, Spain

**Keywords:** Psoriasis, Well-being, Delphi, Quality of life, Skin diseases, Quality of life

## Abstract

Psoriasis is a chronic skin disease that negatively impacts on patient’s life. A holistic approach integrating well-being assessment could improve disease management. Since a consensus definition of well-being in psoriasis is not available, we aim to achieve a multidisciplinary consensus on well-being definition and its components. A literature review and consultation with psoriasis patients facilitated the design of a two-round Delphi questionnaire targeting healthcare professionals and psoriasis patients. A total of 261 panellists (65.1% patients with psoriasis, 34.9% healthcare professionals) agreed on the dimensions and components that should integrate the concept of well-being: emotional dimension (78.9%) [stress (83.9%), mood disturbance (85.1%), body image (83.9%), stigma/shame (75.1%), self-esteem (77.4%) and coping/resilience (81.2%)], physical dimension (82.0%) [sleep quality (81.6%), pain/discomfort (80.8%), itching (83.5%), extracutaneous manifestations (82.8%), lesions in visible areas (84.3%), lesions in functional areas (85.8%), and sex life (78.2%)], social dimension (79.5%) [social relationships (80.8%), leisure/recreational activities (80.3%), support from family/friends (76.6%) and work/academic life (76.5%)], and satisfaction with disease management (78.5%) [treatment (78.2%), information received (75.6%) and medical care provided by the dermatologist (80.1%)]. This well-being definition reflects patients’ needs and concerns. Therefore, addressing them in psoriasis will optimise management, contributing to better outcomes and restoring normalcy to the patient’s life.

## Introduction

Psoriasis is a chronic skin disease that negatively impacts on patients’ quality of life (QoL)^1^. In addition, some psoriasis-associated symptoms may trigger stress^2,3^, low self-esteem^4^ and stigmatisation^5^, impairing the patient’s emotional well-being^6^ and affecting their social^7^ and family relationships^8^ and working capacity^9,10^. Therefore, the disease burden goes beyond the visible nature of the associated psoriatic lesions^1^.

In recent times, therapies have improved significantly in terms of efficacy, safety and tolerability, leading to a reduction in the severity and extent of the disease^11^. However, enhancements in the signs and symptoms of the disease may not have the same impact on the patients’ overall perception of their pathology, their beliefs or coping skills^12^. Hence, it is crucial to develop therapeutic interventions aimed at improving patients’ well-being from a holistic perspective, helping them return to the state of well-being they enjoyed prior to the disease.

Well-being is closely linked to QoL; however, both should be treated as separate concepts. Quality of life refers to the patient’s cognitive assessment of their health’s impact on their daily life^13^. Well-being is a more global and holistic concept that refers to the patient’s emotional response to their illness, treatment, and future^13^. Although related, the two concepts have important differences in approach and assessment. Therefore, the impact of the disease extends beyond its symptoms and signs^14^ and may result in stigmatisation and limitations in social and occupational functioning^15^. The concept of well-being encompasses all areas of life that may be affected by the disease^16^, including aspects that are not usually included in QoL^17^, which tends to focus on three dimensions, physical, mental, social and general health perception^18^.

Integrating well-being in managing diseases, such as psoriasis, where the relationship between psychosocial factors on the occurrence, severity and progression of the disease is well established^19^, could greatly contribute to a better understanding and improved treatment of the disease^20^. In this respect, the Global report on psoriasis published by the World Health Organization (WHO) pointed out the need to assess and consider the full spectrum of the person’s needs, including the issues related to their psoriasis, but also other issues related to their health and well-being^9^. However, there is currently no agreed definition of well-being in the context of psoriasis, making well-being assessment a challenge for patient management.

The incorporation of ‘well-being’ in the WHO’s definition of health as a ‘state of well-being and not merely the absence of disease^21^ has led to a growing body of research on this topic. Consequently, several international institutions, including the WHO, have defined the concept of well-being^22,23^. However, the different definitions, variety of approaches and complexity of the concept have made it difficult to reach an international consensus on how to address and measure it^22–26^. Nevertheless, despite no agreed definition^27^ there is broad agreement that well-being encompasses: mental health, functional status, life satisfaction, personal development, positive social relationships, positive emotions, and the absence of negative emotions^22,23^.

As a starting point to developing a holistic approach to psoriasis, we must reach prior agreement on the definition of well-being in these patients. Therefore, here, we aim to achieve a multidisciplinary consensus, including patients’ perspectives, on defining the concept of well-being in psoriasis and establishing its dimensions and components.

## Materials and methods

A scientific committee consisting of six dermatologists (ED, IB, FG, PC, PDLC, RRV), three hospital pharmacists (JLP, ER, EC), one psychologist (SR) and one patient association representative led the project. Members of the scientific committee were selected based on their experience in managing patients with psoriasis and their particular interest in patients’ well-being and QoL.

This study was performed in line with the principles of the Declaration of Helsinki and informed consent was obtained from all participants.

Due to the nature of the study, which does not collect clinical data from participants, including data on drugs or interventions, and that the questions are related to participants’ perceptions, the approval of an Ethics Committee is not required, as defined in Royal Decree 957/2020 of 3 November^28^ and the Memorandum of Cooperation between Ethics Committees^29^. However, all participants had to read an information sheet and agree to take part in the study to participate.

**Figure 1:**
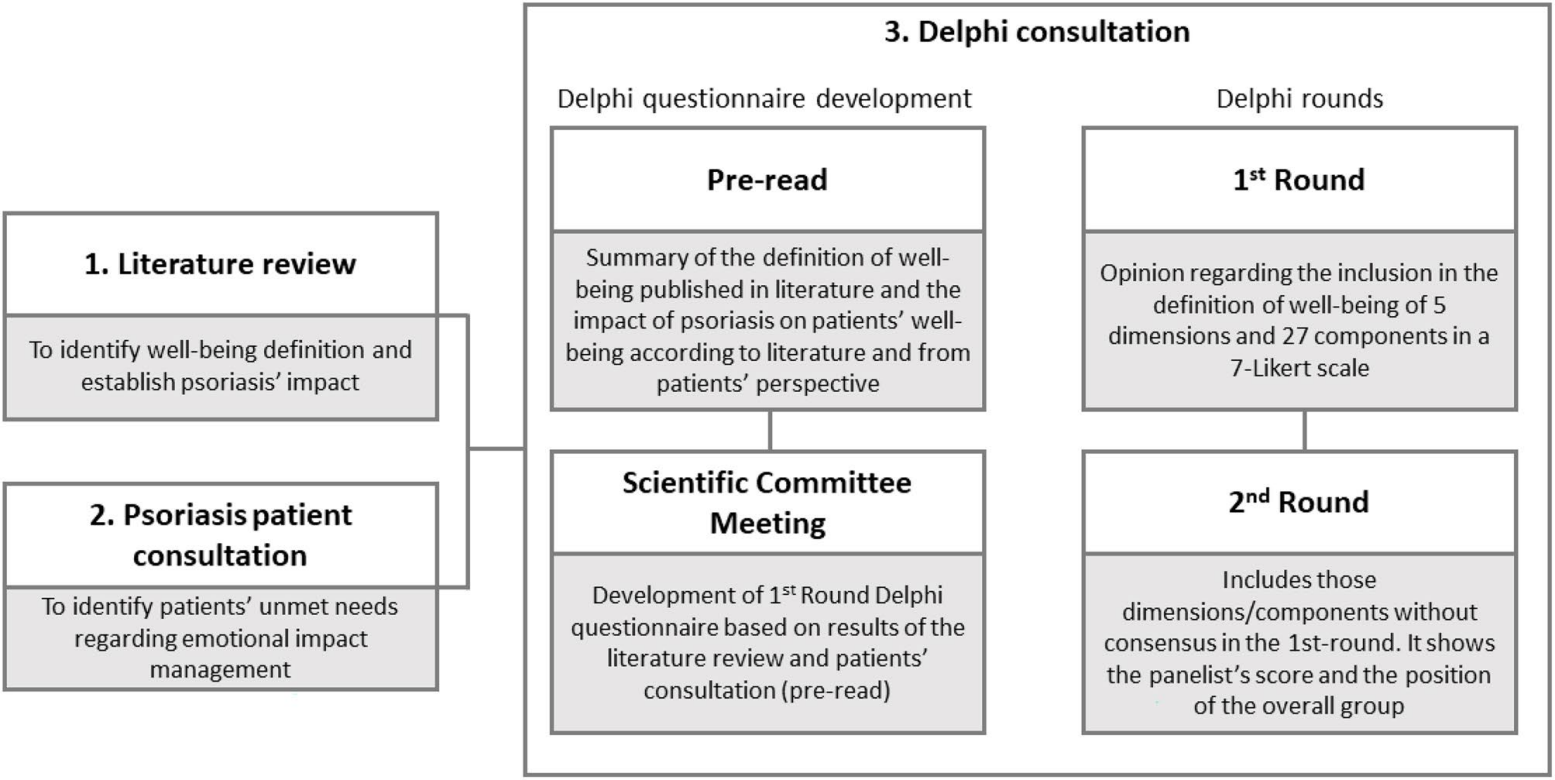
Study phases.

The study comprised three phases: (1) literature review, (2) psoriasis patients’ consultation, and (3) Delphi consultation (Fig. 1).

### Literature review

A literature review in PubMed database was carried out to identify existing definitions of well-being and establish the impact of psoriasis on patients’ well-being and QoL. The international PubMed database was consulted using MeSH terms and open terms combined with the Boolean operators “OR”, “AND”, or “NOT”. We used terms related to well-being [wellbeing, well-being, personal satisfaction (MeSH), life satisfaction, personal satisfaction], measurement instruments [health surveys (MeSH), questionnaire, self-assessment, scale, instrument, index, measure, tool], psoriasis [psoriasis (MeSH), psoriasis] and areas where it can have an impact (stress, emotional, anxiety, depression, suicide, suicidality, cognitive, impairment, body image, stigma, self-esteem, coping, psychosocial coping, sleep quality, sleep disorders, work productivity, pain, discomfort, itching, pruritus, symptoms, social, sexual, treatment satisfaction, life satisfaction, social and sexual health).

The search included publications in English or Spanish without a limited period (but the most recent articles were prioritised). Articles referring to patients with psoriatic arthritis exclusively or associated with a specific treatment were not reviewed. In addition, a search of the grey literature [Google Academic, WHO website (www.who.int), patient association websites (www.accionpsoriasis.org and www.psoriasis.org), dermatological societies’ websites (www.aedv.es and www.eadv.org)] was also performed using terms related to well-being and psoriasis.

### Psoriasis patient consultation

Through the patient association “*Acción psoriasis*” and with the active support of the patient community, an online patient survey addressed to 865 patients with psoriasis was conducted in November 2021 to identify the main unmet needs regarding the management of the emotional impact of psoriasis (details will be published elsewhere).

The survey included questions about the relationship between psoriasis and emotional health, the implications of psoriasis in patients’ lives, and the emotional management of psoriasis (information and care provided by healthcare professionals to address psychological needs).

### Delphi consultation

The Delphi technique is a formal and systematic method for obtaining expert consensus on a particular knowledge area. Its main advantages include its high capacity to integrate information, identify positions of groups and individuals, and determine or order priorities^30^. It is an iterative process characterised by the anonymity of participants, controlled feedback of response and the statistical aggregation of group responses^31,32^.

The Delphi methodology does not have a set minimum number of panellists, as the decision is based on empirical and pragmatic factors. The qualities of the expert panel are more important than the number of panellists^32^. On average, Delphi consultations tend to have a sample size between 6 and 50^33^.

Two rounds of Delphi consultation were carried out using an electronic questionnaire. These rounds were considered sufficient to obtain consensus, prevent participant fatigue and reduce the number of non-responses^34^. The 1st-round questionnaire was available from 25 July to 16 September 2022, while the 2nd-round took place from 3 October to 9 November 2022.

#### Delphi questionnaire

The scientific committee developed the Delphi questionnaire based on the literature review results and the consultation with psoriasis patients.

The 1st-round Delphi questionnaire encompassed statements regarding the dimensions (n = 5) and components (n = 27) that integrate the definition of well-being (Table 1).

**Table 1:** Dimensions and components of well-being included in the 1st-round of the Delphi questionnaire.

	Dimension				
	Emotional	Physical	Social	Satisfaction with disease management	Personal development
Components	Stress (or distress)	Quality of sleep	Social relations	Satisfaction with treatment	Personal development
	Mood disorder (anxiety/depression)	Physical fitness	Leisure/Recreational activities	Satisfaction with information received	Career development
	Body image	Pain/discomfort	Support from family/friends	Satisfaction with the medical care provided by the dermatologist	
	Stigmatisation or shame	Itching	Work/academic life	Satisfaction with the care provided by other health professionals	
	Self-esteem	Extracutaneous manifestations	Social rejection	Satisfaction with public/private administration	
	Coping/resilience	Lesions visible areas			
		Lesions in functional areas			
		Sex life			
		Cognitive impairment			

Panellists’ opinion on each dimension/component was assessed using a 7-point Likert scale (1, strongly disagree; 2, mostly disagree; 3, somewhat disagree; 4, neither agree nor disagree; 5, somewhat agree; 6, mostly agree; 7, strongly agree).

Panellists also specified their level of agreement with the proposed definition of well-being (n = 1) and with the two options of descriptors suggested for each dimension (n = 5).

The questionnaire also included a free-text space where panellists could make observations and comments.

The 2nd-round questionnaire covered those dimensions/components for which consensus was not reached in the 1st-round. It was specifically tailored to each panellist, showing the panellist’s score and the position of the overall group (range of the greatest percentage of scores). In addition, it included two definitions of well-being (developed from the descriptors evaluated in the 1st-round) to be assessed by the panellists.

#### Delphi panellists

Delphi questionnaires were addressed to healthcare professionals, experts in psoriasis management and psoriasis patients who are members of the Spanish patient association (*Acción Psoriasis*). The Scientific Committee did not participate in the Delphi rounds. Healthcare professionals were selected and invited to participate through the study sponsor, while psoriasis patients were identified and invited to participate by the patient association.

#### Consensus definition

The consensus definition was established for each statement before data analyses, according to the common criteria^35^. Consensus was reached when at least 75% of the respondents agreed (strongly and mostly agree: 6, 7) or disagreed (strongly and mostly disagree: 1, 2) with dimension/component inclusion.

### Data analysis

The descriptive analysis of panellists’ characteristics [mean and standard deviation (SD)] and the percentage of participants who selected each option (disagree: 1, 2; neutral: 3, 4, 5; agree: 6, 7) was conducted using STATA statistical software, V.14.

### Ethics statement

Due to the nature of the study, an Ethics Committee approval is not required as clinical data, including medication or intervention data, is not collected and only participants’ perceptions are collected. However, all participants were required to read an information sheet and provide their consent to participate in the study.

## Results

A total of 261 panellists participated in the Delphi consultation: 170 (65.1%) psoriasis patients [62.94% women, mean age 48.51 (SD 10.62) years, mean years from diagnosis 24.16 (SD 13.27)] and 91 (34.9%) healthcare professionals [56.04% women, mean age 48.42 (SD 10.08), mean years of experience 21.23 (SD 10.49), 66% dermatologists with experience in psoriasis or members of the Spanish Academy of Dermatology and Venereology (AEDV) Psoriasis Group, 34% hospital pharmacists]. The response rate in the 2nd-round was 99% among health professionals and 85% among patients.

### Dimensions of well-being

**Figure 2:**
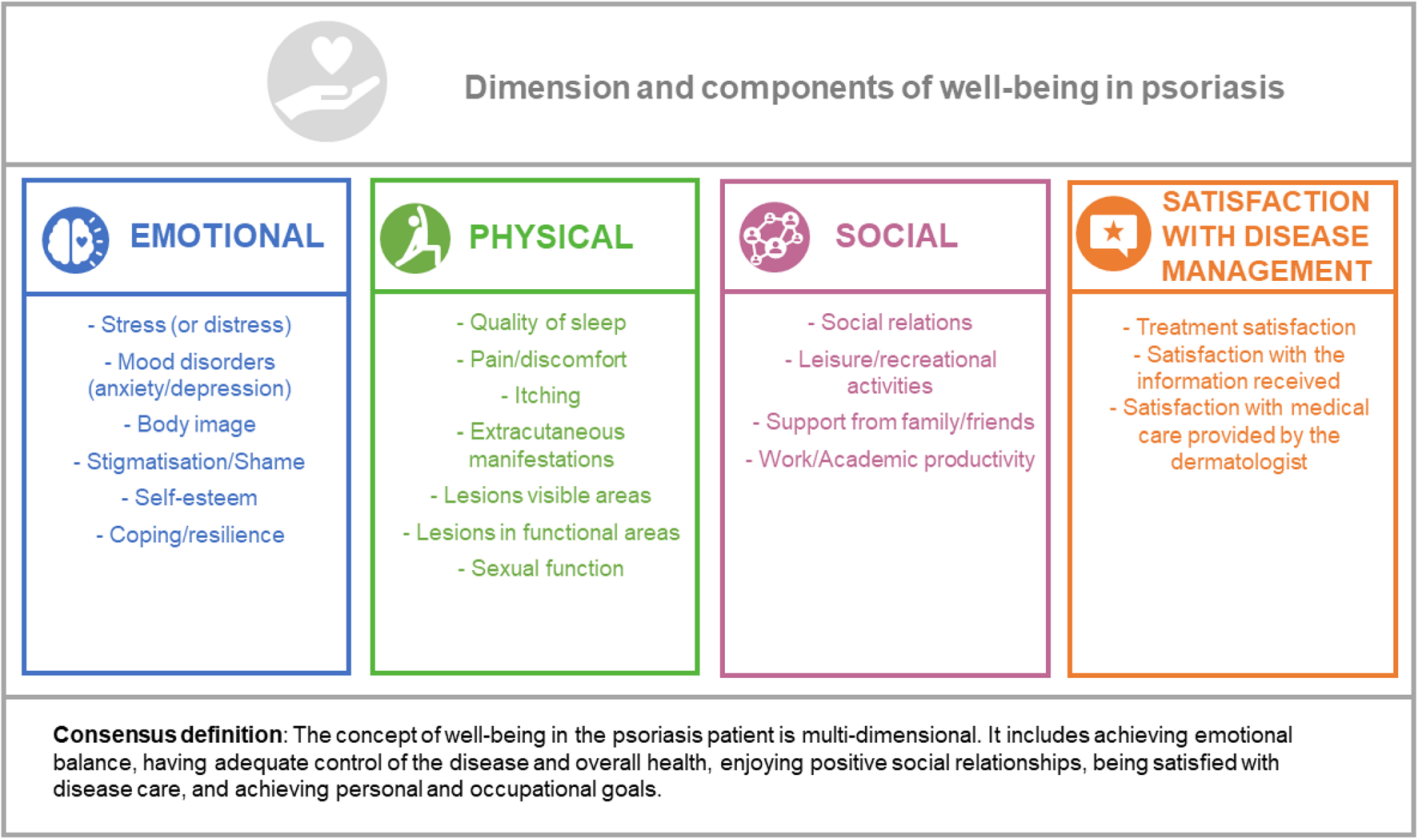
Dimensions and components of well-being in psoriasis.

The panellists agreed that the concept of well-being was integrated by emotional, physical, social and satisfaction with disease management dimensions. However, the personal development dimension did not reach a consensus for its inclusion (Table 2, Fig. 2).

**Table 2:** Results of Delphi consultation: Well-being dimensions.

Dimension	Disagree	Neutral	Agree	Round
The concept of well-being in patients with psoriasis is multi-dimensional and includes the **Emotional** dimension	23 (8.8%)	32 (12.3%)	**206 (78.9%)**	1
The concept of well-being in patients with psoriasis is multi-dimensional and includes the **Physical** dimension	17 (6.5%)	30 (11.5%)	**214 (82.0%)**	1
The concept of well-being in patients with psoriasis is multi-dimensional and includes the **Social** dimension	21 (9.0%)	27 (11.5%)	**186 (79.5%)**	2
The concept of well-being in patients with psoriasis is multi-dimensional and includes **Satisfaction with disease management** dimension	20 (7.7%)	36 (13.8%)	**205 (78.5%)**	1
The concept of well-being in patients with psoriasis is multi-dimensional and includes the **Personal development** dimension	24 (10.3%)	66 (28.2%)	144 (61.5%)	2

Bold, dimensions that reached agreement (≥ 75%).

#### Components of the emotional dimension

The panellists agreed that the emotional dimension comprised stress (or distress), mood disorders, body image, stigmatisation/shame, self-esteem, and coping/resilience (Table 3, Fig. 2).

**Table 3:** Results of Delphi consultation: components of well-being dimensions.

	Disagree	Neutral	Agree	Round
Emotional dimension				
Stress (or distress)	17 (6.5%)	25 (9.6%)	**219 (83.9%)**	1
Mood disorders (anxiety/depression)	17 (6.5%)	22 (8.4%)	**222 (85.1%)**	1
Body image	14 (5.4%)	28 (10.7%)	**219 (83.9%)**	1
Stigmatisation or shame	16 (6.1%)	49 (18.8%)	**196 (75.1%)**	1
Self-esteem	19 (7.3%)	40 (15.3%)	**202 (77.4%)**	1
Coping strategies/resilience	13 (5.0%)	36 (13.8%)	**212 (81.2%)**	1
Physical dimension				
Sleep quality	13 (5.6%)	30 (12.8%)	**191 (81.6%)**	2
Physical fitness	15 (6.4%)	46 (19.7%)	173 (73.9%)	2
Pain/discomfort	14 (5.4%)	36 (13.8%)	**211 (80.8%)**	1
Itching	16 (6.1%)	27 (10.3%)	**218 (83.5%)**	1
Extracutaneous manifestations	14 (5.4%)	31 (11.9%)	**216 (82.8%)**	1
Lesions visible areas	16 (6.1%)	25 (9.6%)	**220 (84.3%)**	1
Lesions in functional areas	15 (5.8%)	22 (8.4%)	**224 (85.8%)**	1
Sex life	8 (3.4%)	43 (18.4%)	**183 (78.2%)**	2
Cognitive impairment	18 (7.7%)	92 (39.3%)	124 (53.0%)	2
Social dimension				
Social relationships	11 (4.7%)	34 (14.5%)	**189 (80.8%)**	2
Leisure/recreational activities	11 (4.7%)	35 (15.0%)	**188 (80.3%)**	2
Friends and family support	12 (4.6%)	49 (18.8%)	**200 (76.6%)**	1
Working/academic life	8 (3.4%)	47 (20.1%)	**179 (76.5%)**	2
Social rejection	13 (5.6%)	49 (20.9%)	172 (73.5%)	2
Satisfaction with disease management dimension				
Satisfaction with treatment	16 (6.1%)	41 (15.7%)	**204 (78.2%)**	1
Satisfaction with information received	9 (3.6%)	48 (20.5%)	**177 (75.6%)**	2
Satisfaction with the medical care provided by the dermatologist	15 (5.8%)	37 (14.2%)	**209 (80.1%)**	1
Satisfaction with the care provided by other health professionals	11 (4.7%)	52 (22.2%)	171 (73.1%)	2
Satisfaction with public/private health administration	13 (5.6%)	13 (5.6%)	161 (68.8%)	2
Personal development dimension				
Personal development	9 (3.9%)	54 (23.1%)	171 (73.1%)	2
Career development	9 (3.9%)	68 (29.1%)	157 (67.1%)	2

Bold, components that reached agreement (≥ 75%).

#### Components of the physical dimension

The panellists agreed that the physical dimension consisted of the following components: sleep quality, pain/discomfort, itching, extracutaneous manifestations, lesions in visible areas, lesions in functional areas, and sex life. However, no consensus was reached on physical fitness or cognitive impairment (Table 3, Fig. 2).

#### Components of the social dimension

The panellists reached consensus that the social dimension was formed by: social relations, leisure/recreational activities, support from family/friends, and work/academic life and integrated the social dimension. However, no consensus was reached on social rejection (Table 3, Fig. 2).

#### Components of the satisfaction with disease management dimension

The panellists agreed that satisfaction with treatment, satisfaction with the information received, and satisfaction with the medical care provided by the dermatologist were components of the satisfaction with disease management dimension. However, no consensus was reached on satisfaction with the care provided by other health professionals and satisfaction with public/private administration (Table 3, Fig. 2).

#### Components of the personal development dimension

No consensus was reached on including any of the proposed components of the personal development dimension (Table 3).

### Definition and descriptors for each dimension

Panellists agreed on the following definition of well-being: “*The concept of well-being in the psoriasis patient is multi-dimensional. It includes achieving emotional balance, having adequate overall health and control of the disease, enjoying positive social relationships, and being satisfied with disease care*” (Table 4).

**Table 4:** Results of Delphi consultation: definition and descriptors of well-being.

Well-being definition				
The concept of well-being in the psoriasis patient is multi-dimensional. It includes achieving emotional balance, having adequate overall health and control of the disease, enjoying positive social relationships, being satisfied with disease care, and achieving personal and career goals				**160 (70.5%)**
The concept of well-being in the psoriasis patient is multi-dimensional. It includes enjoying good mental health, having adequate overall health and control of the disease, having family/social support and positive social relationships, being satisfied with disease management and feeling personally and professionally fulfilled				69 (29.5%)

Descriptors	Option 1	n (%)	Option 2	n (%)
Emotional dimension	Achieve emotional balance	105 (40.2%)	Enjoy good mental health	**156 (59.8%)**
Physical dimension	Have adequate overall health and control of the disease	**173 (66.3%)**	Be in good health and have no disease-related symptoms	88 (33.7%)
Social dimension	Enjoy positive social relationships	56 (21.5%)	Have family/social support and positive social relationships	**205 (78.5%)**
Satisfaction with disease management dimension	Be satisfied with disease care	55 (21.1%)	Be satisfied with disease management	**206 (78.9%)**
Personal development	Achieve personal and work goals	44 (16.9%)	Feel personally and professionally fulfilled	**217 (83.1%)**

Bold, definitions and descriptors that reached agreement (≥ 75%).

## Discussion

To our knowledge, this is the first study aimed at defining well-being in psoriasis. Psoriasis is a chronic disease with a high physical and psychological impact on patients^36,37^; hence, establishing the definition of well-being in the context of psoriasis can help patients and healthcare professionals to better understand the disease, promoting a holistic approach to its management. The multidisciplinary nature of the consensus achieved, incorporating both healthcare professionals and patients with psoriasis, has enabled us to obtain an integrated and comprehensive definition of well-being.

The emotional dimension and all its components reached consensus in the 1st-round, highlighting the high impact of psoriasis on this dimension. Previous studies have shown the emotional impact of psoriasis, which supports the inclusion of this dimension in the definition of well-being. Stress has been identified as one of the symptoms triggering the disease and is an exacerbating factor^2,38^; and research shows an association between psychological stress and the onset^2^, recurrence and severity of psoriasis^3,38^. In addition, other emotional disorders, such as anxiety or depression, are common in this population. For example, a previous study conducted in Spain using the Hospital Anxiety and Depression Scale (HADS) questionnaire indicates that 40% and 38% of patients with psoriasis suffer from depression and anxiety, respectively^39^. Patients with therapy-controlled psoriasis are psychologically affected, and despite improvement in their skin lesions, a high percentage of patients still have symptoms of anxiety or depression^36,40^. Therefore, besides enhancing the patient’s skin lesions, psychological care is also necessary because the psychological impact may remain^36^. Mental health assessment is currently a crucial aspect of psoriasis patient care. Efforts are being made to optimise clinical practice by informing patients about this relationship, assessing the most appropriate treatment, and referring them to a specialist when necessary^41,42^.

The emotional dimension can be influenced by self-image and self-esteem, especially when psoriasis affects visible body areas. The visibility of psoriatic lesions may also cause fear, disquiet, rejection or embarrassment, contributing to the stigmatisation of patients^5^. Therefore, some studies suggest that patients with psoriasis are less satisfied with their body image than the general population^43,44^ and have lower self-esteem^4^. Thus, using effective coping strategies and training patients with lower health related quality of life to use these coping strategies is necessary to reduce the high impact of the illness on patients’ well-being^45^.

The physical dimension is also considered essential in the definition of well-being. Symptoms such as pain/discomfort, itching and scaling are common in patients with psoriasis and are considered the most bothersome^46^. In addition, these symptoms have a negative impact on the patient’s well-being, affecting their daily life, quality of sleep, mood, stigmatisation, and life satisfaction^47,48^. Sleep quality and sexual function are also affected by psoriasis. Previous studies show that patients with psoriasis have poorer sleep quality than the general population^49^ and have a higher prevalence of sleep disorders such as obstructive sleep apnoea and restless legs syndrome^50^. In addition, there is evidence that symptoms of insomnia in psoriasis are directly mediated by pruritus and pain^50^, so treatments that decrease the cutaneous symptoms in psoriasis successfully mitigate insomnia^50^. However, there was no consensus on the physical fitness component and cognitive impairment. Regarding physical fitness, psoriasis patients may have reduced willingness to engage in physical fitness due to lesions in functional areas and itching which can impact their motivation and overall physical fitness^51^. Although many respondents considered this component to be important, there was no consensus. It is worth nothing that the main causes of discomfort during physical activity, such as lesion location and pruritus, have achieved consensus. Some panellist may have interpreted physical fitness as a secondary consequence of these components.

Regarding cognitive impairment, several studies suggest a potential relationship between psoriasis and cognitive impairment, although the results are heterogeneus^52,53^. Hoiwever, this association seems to be stronger in patients with severe psoriasis. Althougt this component was considered relevant by many penellist, it did not reach consensus and was therefore left out of the definition.

Regarding sexual function, due to severity, pain or embarrassment in the case of genital implication^54^, both men and women with psoriasis are at increased risk of sexual dysfunction^55^. There was no consensus on the inclusion of the dimension or components of personal development in the concept of well-being, so it was left out of the definition. It is important to note that patients with psoriasis have achieved personal development thanks to improvements in current treatments and therapies. This means that the disease does not currently impede the personal and professional development of the patients.

Consensus was also reached on the inclusion of the social dimension in the definition of well-being. Panellists agreed that family support/friendships, social relationships, leisure/recreational activities, and work/activity were components of the social dimension. Previous studies indicate that psoriasis negatively impacts social interaction skills, making it challenging to make new friends, avoiding social engagements or modifying how one dresses^56^. In addition, it also affects work productivity, which is directly correlated with the severity of the disease^10^.

Fortunately, today’s ease of access to information about the disease by the general population has contributed to reducing the belief that it is a contagious disease, along with discrimination and trivialisation of the illness. This could have reduced the social rejection felt by patients, which may explain why the social rejection component did not reach a consensus among the panellists.

The dimension of satisfaction with disease management also reached consensus, as did the component’s satisfaction with treatment, with the care provided by the dermatologist and the information received. A recent study indicates that psoriasis patients’ dissatisfaction with the treatment received is very high (more than 50%)^57^; moreover, 40% of patients desire more effective therapies^58^. In contrast, consensus was not reached on satisfaction with the public/private administration and the care provided by other health professionals. In Spain, there is less interaction with other professionals, such as nurses or psychologists, with the dermatologist being the primary healthcare provider for these patients.

The description of well-being in patient with psoriasis, as agreed upon in this study, will enable healthcare professionals to assess the impact of each dimension in the patient with psoriasis, and determine what is the most important to them. Defining well-being can help healthcare professionals to understand well-being and achieve a patient-centred care.

In line with our results, a recent nationwide survey conducted in Italy aimed to identify key factors contributing to barriers impacting patients’ well-being, which indicated that although the physical burden experienced by patients with moderate-to-severe psoriasis is significant, non-physical domains such as social and mental areas are also impacted to a similar extent^16^. Similarly, van Ee’s et al.^59^ recently published a definition of ‘freedom from disease’ including five domains: QoL and well-being, healthcare team support, treatment, psychosocial elements, and management of clinical symptoms.

The present study presents several limitations inherent to the Delphi methodology. Firstly, the inclusion of patients with psoriasis on the Delphi panel may be limiting, despite also representing one of the study’s main strengths. Patients included in the Delphi panel were members of the Spanish patient association (*Acción Psoriasis*), and therefore they may have an extensive history and strong awareness of their disease. In addition, the Delphi methodology may be challenging for some patients, positioning some of them in the neutral zone. Secondly, even though the consensus threshold was established at the beginning of the study according to recommendations, using another cut-off could give rise to different results. Finally, the study was conducted in Spain; therefore, although the concept of well-being is universal and no major differences are expected in other settings, the results should be interpreted in the Spanish context.

In conclusion, the concept of well-being in psoriasis integrates four dimensions, emotional, physical, social and satisfaction with disease management. All these dimensions and their components reflect patients’ needs and concerns that impact on their daily lives. Therefore, addressing well-being components in psoriasis management will optimise it, contributing to obtaining better outcomes and restoring normalcy to the patients’ life. Future work could evaluate the weight of each of the components and assess differences based on variables.

### Supplementary Information

Supplementary Information.

## Data Availability

The original contributions presented in the study are included in the article/Supplementary Material. Further inquiries can be directed to the corresponding author.
