# Supplementary material for: Defining well-being in psoriasis: A Delphi consensus among healthcare professionals and patients
Source: Sci Rep. 2024 Jun 24;14:14519. doi: 10.1038/s41598-024-64738-6 (PMC11196587; doi:10.1038/s41598-024-64738-6)
Supplement: Supplementary file 1 — Supplementary Information. [file 41598_2024_64738_MOESM1_ESM.docx]

**Supplementary Material**

**Stress (or distress):** a feeling of physical or emotional tension resulting from any change in our lives, positive or negative, involving an overload that continues over time, which can generate feelings of nervousness, worry, irritability, difficulty to concentrate and exhaustion.

**Mood disorders** (depression/anxiety): emotional disturbances characterised by anxious and/or depressive symptoms in which the person presents a low mood, loss of interest in daily activities (anhedonia) or excessive worry and nervousness (anxiety) that interfere with daily life.

**Body image / body self-perception:** Body image is the mental representation that each person has of his or her own physical appearance. It is how the person sees him/herself and how he/she perceives him/herself when looking in the mirror, in other words, what the person thinks he/she is like. Body image also has to do with the feelings and emotions a person experiences about how they perceive their physique, how they feel about their body and within their own body.

**Stigmatisation:** negative association between a person or a group of people who share certain characteristics or a certain disease. Stigmatisation can lead to these people being labelled, stereotyped, discriminated against or rejected because of this condition**.**

**Shame:** is the social emotion that arises when we value our actions as negative, i.e., we believe we are doing something wrong and that this will lead others to make negative judgements about us. It is a very common type of emotion in society: shyness, blushing, embarrassment or withdrawal**.**

**Self-esteem:** Self-esteem is the set of beliefs, perceptions, evaluations and thoughts we have about ourselves, the evaluation we make based on our experiences. Self-esteem can be a positive or negative evaluation of oneself. It is the predisposition to know oneself to be fit for life and to satisfy one's own needs, to feel competent to face the challenges that arise and deserving of happiness.

**Coping strategies:** refer to efforts, through overt or internal behaviour, to cope with internal and/or environmental demands that exceed the person's resources**.** They include those cognitive and behavioural strategies that the person uses to solve, cope with or manage a particular problem or stressful event.

**Resilience:** the ability to adapt to an adverse situation or state.

**Sleep quality:** good sleep quality involves both a subjective assessment of rest, as well as the quantitative dimension (sleep duration, sleep latency, number of nightly awakenings) and the purely subjective qualitative dimensions (depth of sleep or its restoration capacity).

**Physical fitness:** to be in good physical shape so as to perform physical activity that is not interfered with by the disease.

**Pain:** a localised, subjective, sensory perception of varying intensity, which may be uncomfortable or unpleasant in a part of the body.

**Discomfort:** physical sensation of general and vague indisposition and discomfort**.**

**Itching:** a sensation of itching, prickling, peculiar tingling or uncomfortable irritation of the skin that causes the need or desire to scratch.

**Extracutaneous manifestations:** extension of psoriasis beyond the skin: joints (psoriatic arthritis), digestive system (Crohn's disease, ulcerative colitis), obesity, among others.

**Lesions in visible areas:** presence of psoriasis plaques on the face, scalp, hands and arms.

**Lesions in functional areas:** presence of psoriasis plaques on the palms of the hands, nails, soles of the feet or joints, which hinder the individual's functional ability**.**

**Sex life:** encompasses those behaviours that concern sexual pleasure and the satisfaction of sexual desire.

**Cognitive impairment (attention or memory deficits)**: includes impairment of one or more cognitive functions such as processing or recalling information, attention, language or memory.

**Social relationships:** set of interactions that occur between two or more people according to a set of specific conventions or rules. They include affective, work, family and circumstantial relationships.

**Leisure/recreational activities:** activities that are carried out in free time and are not related to work or household chores. They include those pastimes that the individual has chosen voluntarily, and which he/she can organise as he/she wishes**.**

**Friends and family support:** togetherness, communication, trust, cohabitation and the support existing between family members or friends.

**Working life:** set of manual or intellectual activities performed by an individual in exchange for economic compensation for the work done.

**Academic life:** set of activities aimed at academic training (intellectual growth that helps the individual to develop analytical and critical skills, while also being prepared for problem solving).

**Social rejection:** when a person or group of people are deliberately excluded from a relationship or social interaction.

**Satisfaction with treatment:** the degree to which the treatment process and the results obtained have met the patient's expectations and needs.

**Satisfaction with information received:** the degree to which the information received about the disease and its treatment has met the patient's expectations and needs.

**Satisfaction with medical care provided by the dermatologist:** degree to which the healthcare provided by the dermatologist has met the patient's expectations and needs.

**Satisfaction with the care provided by other healthcare professionals:** the degree to which the healthcare provided by the healthcare professionals (medical staff, nursing staff, pharmacy) responsible for the patient's care has met the patient's expectations and needs.

**Satisfaction with the public/private health administration:** the degree to which the health services provided by the public/private health system have met the patient's expectations and needs.

**Personal development:** individual growth that fosters the development of personal skills and/or abilities for the achievement of life goals and objectives.

**Career development:** occupational improvement or growth based on the development of personal skills, leading to the achievement of goals, objectives and improvements in the workplace.
